# From the WHO framework to integrated senior health and wellness hub program: an implementation journey

**DOI:** 10.3389/fpubh.2025.1593490

**Published:** 2025-07-28

**Authors:** Xiaoting Huang, Hao Yi Tan, Pei Ling Er, Amber Wong, Si Qi Lim, Joshua Kuan Tan, Wan Qi Yee, Xiaohui Xin, Lian Leng Low

**Affiliations:** ^1^Population Health and Integrated Care Division, Singapore General Hospital, Singapore, Singapore; ^2^Department of Geriatric Medicine, Singapore General Hospital, Singapore, Singapore; ^3^Health Services Research Unit, Singapore General Hospital, Singapore, Singapore; ^4^Health Systems Group, Harvard T.H. Chan School of Public Health, Boston, MA, United States; ^5^Practice Office, Saw Swee Hock School of Public Health, Singapore, Singapore; ^6^Department of Dietetics, Singapore General Hospital, Singapore, Singapore; ^7^Department of Physiotherapy, Singapore General Hospital, Singapore, Singapore

**Keywords:** aging in place, seniors, Theory of Change, consolidated framework for implementation research, ICOPE

## Abstract

**Introduction:**

As global populations age, ensuring older adults maintain functional ability and wellbeing is a critical public health priority. The Integrated Senior Health and Wellness (ISHW) Hub program, developed by Singapore General Hospital (SGH), operationalizes the World Health Organization’s (WHO) Integrated Care for Older People (ICOPE) framework within community-based models of care. Implemented in partnership with Active Aging Centers (AACs), ISHW enhances preventive health through structured screening, customizable nutrition education, and targeted physical activity interventions.

**Methods:**

This study aimed to develop an implementation-informed, context-sensitive model for integrated care using the Consolidated Framework for Implementation Research (CFIR) and Theory of Change to guide the design and pilot of the ISHW program. The ISHW program employs a multi-prong approach, integrating health interventions with social engagement to address both physical and psychosocial determinants of health. Key components include community-driven intrinsic capacity assessment including chronic disease screening for early detection, dietitian-curated nutrition education and exercise program enhanced by physiotherapist. A critical innovation is the “train-the-trainer” model, which enhances capacity-building among AAC staff and volunteers which supports sustainable program delivery.

**Results:**

Qualitative insights were gathered from 5 focus groups (*n* = 20) and 1 feedback group (*n* = 10), analyzed thematically through consensus-driven coding. Early implementation findings highlight the feasibility of integrating ICOPE principles within community-based settings, but also underscore key challenges, including resource constraints and the need for stronger inter-sectoral coordination. Facilitators of success include structured capability-building, community participation, and alignment with national aging policies such as Age Well SG and Healthier SG. Findings gleaned from CFIR and ToC was operationalized to a program’s logic model that was iteratively refined to enhance adaptability and scalability across diverse community settings.

**Discussion:**

This case study adds to current understanding of community-centered models for promoting healthy aging and helps to serve as a guidance on how ICOPE-based program could be adapted to other community settings for integrating health and social care to support aging in place. Future evaluations will assess longitudinal outcomes and inform the scalability of community-based ICOPE implementation strategies.

## Introduction

### The challenge of healthy aging

As global life expectancy increases, it is critical to ensure that older persons maintain their functional ability and wellbeing. The United Nation’s Decade of Healthy Aging (1) initiative underscores the importance of age-friendly communities and integrated care. Implementing the Integrated Care for Older Persons (ICOPE) framework as a part of Universal Health Coverage is one of the action areas of UN Decade of Healthy Aging, with ICOPE focusing on improving the functional ability and intrinsic capacity of older adults through person-centered, coordinated models of care.

The ICOPE (2) care pathway framework comprises five essential steps: screening, comprehensive assessment, intervention, monitoring, and community public health action. It aims to enhance intrinsic capacity by integrating nutrition, physical activity, and social engagement into care plans. However, existing literature on implementing ICOPE highlights several challenges. One significant issue is the limited capacity for consistent and comprehensive screening outside of clinical settings (3). Moreover, there is often a shortage of trained personnel and resources for conducting thorough assessments of older adults (4). Coordination between healthcare and social services is also often lacking (4), making it difficult to create integrated care plans.

### The ISHW program as an innovative response

In response to these challenges, Singapore has actively engaged in international collaborations (5) to learn from best practices in implementing the ICOPE framework. The Integrated Senior Health and Wellness (ISHW) program developed by Singapore General Hospital (SGH), leverages the ICOPE framework to enhance preventative health and integrated care for seniors. Targeting community dwelling older adults aged 60 and above, healthcare professionals from SGH collaborate with Active Aging Centers (AAC), centers located in residential areas in Singapore serving older adults aged 60 and above stay in designated blocks in the community (6), to deliver the program. The program aims to grow existing AACs into ISHW Hubs that serve as comprehensive, one-stop centers supporting healthy and active aging, and foster social connections. Through integrating healthcare interventions with social engagement opportunities, the ISHW program promotes physical wellbeing while addressing issues of social isolation among seniors.

#### Key tenets of the ISHW program

The ISHW Program is structured around three core components—screening, nutrition, and physical activity—to address the health and social needs of older adults. Together, these elements form a coordinated and sustainable model of care aligned with WHO’s ICOPE framework. The overall program had been curated based on the expertise of clinicians from the Geriatric Medicine Department, Physiotherapy and Dietetics.

#### Screening

Screening is the first fundamental component of the program, meant to allow community providers to identify health conditions and behaviors that may impact older adults’ wellbeing using a structured questionnaire adapted from WHO’s ICOPE screening questionnaire. Under the program, each participant receives a personalized health report with recommendations for various health domains screened based on their screening results. Residents with at higher health needs were directed to follow up by community nurses or trained healthcare workers while those with lower health needs were directed to general health education interventions.

#### Nutrition

Nutrition is key to ISHW program because of its central role in supporting health, and at the same time food can provides a communal platform for social interaction. Working with AACs, ISHW Program looked to complement existing communal dining initiatives with nutrition education, whereby SGH dietitians would equip AAC staff and volunteers with knowledge and skills to promote healthy eating habits among seniors.

#### Physical activity

Thirdly, looking at maintaining physical function of community-dwelling older adults, promoting physical activity is the final component to ISHW’s multi-component approach. The program looked at enhancing group exercises conducted at AACs based on existing guidelines on exercise and rehabilitation, involving review of existing exercise programs through a standardized checklist and upskilling of AAC staff and volunteers by SGH physiotherapists.

### Impact and reach of ISHW program

From January 2023 to December 2024, the ISHW program had extended our reach to six AACs, with key activities piloted at two AACs and slated to be scaled to the remaining AACs We also provided community-based integrated screening to a total of 2,114 older adults residing in the southeast region of Singapore, where 1,624 older adults were provided intervention after initial screening. By end of 2024, we had trained a total of 347 community care providers and volunteers.

## Aims

This study aimed to design and pilot a context sensitive program informed by the Consolidated Framework for Implementation Research (CFIR) (7) and Theory of Change (ToC) (8) methodologies to identify key determinants and mechanisms underpinning the successful implementation of the ISHW program. Considering challenges such as limited system capacity, workforce training deficiencies, and fragmented integration between health and social care sectors, CFIR and ToC were applied to elucidate critical implementation facilitators and co-develop a comprehensive, theory-informed logic model.

This paper follows a stepwise, implementation science–informed approach to outline and present insights gather from development of ISHW Program. First, we use CFIR to highlight key contextual factors, stakeholder needs, and system-level enablers and barriers that had influence program development and pilot implementation. To unveil the workings of the program, the program’s intended outcomes, causal pathways and underlying assumptions will be articulated through a ToC. The program ToC is subsequently operationalized into a logic model, which defined specific inputs, activities, outputs, and outcomes in measurable terms. This structured model will be used to guide the program’s scaled up implementation across Active Aging Centers (AACs) in Singapore.

Beyond the current paper, the authors plan to carry out a subsequent study to evaluate the effectiveness and impact of the program, thus contributing to a growing evidence base on the viability of community-driven health and wellness initiatives for aging populations.

### Study design

Information on the program were drawn from existing program activities as well as structured workshops with program team members. Existing program activities included separate focus group discussions with residents to understand needs (*n* = 20) and obtain program feedback (*n* = 10), as well as meeting documents from program team’s engagement with various stakeholders. This paper is part of the larger evaluation study of ISHW program, which was approved by the SingHealth Centralized Institutional Review Board (CIRB) under reference number 2024–3826 for waiver of documentation of consent. Verbal consent for focus group discussions was collected, with no identifiable data was collected. Future quantitative analyses also utilize secondary deidentified data.

A completed Standards for Reporting Qualitative Research (SRQR) checklist detailing section-by-section coverage is available in Supplementary Appendix 1.

## Context of innovation

### Contextualizing ISHW program using consolidated framework of implementation research framework

A multidisciplinary CFIR mapping team (comprising of one geriatrician, one dietitian, one physiotherapist, one health service researcher, one program executive representing the AACs) conducted three structured two-hour mapping sessions. Using a code-book thematic analysis approach, each member independently assigned CFIR constructs to field-notes in Excel, discussed discrepancies line-by-line, and reached negotiated consensus. The consolidated Excel sheet served as the shared code-book for subsequent phases of analysis.

#### Outer setting

The development and early implementation of the ISHW program were significantly influenced by contextual factors within the outer setting domain, particularly national policies, financing structures, and community expectations. The program’s strategic alignment with Singapore’s broader health priorities and the United Nations Decade of Healthy Aging positioned it advantageously within the policy environment.

Two major national initiatives, Healthier SG (9) and Age Well SG (10), launched during the pilot phase, served as critical external facilitators. Healthier SG, initiated in July 2023, marked a systemic shift toward preventive health by emphasizing early screening, chronic disease management, and community-level health promotion. Subsequently, Age Well SG promoted an age-friendly ecosystem integrating health, social, and environmental supports.

These initiatives imposed external mandates and performance expectations that exerted pressure on community-based providers, including Active Aging Centers (AACs), to expand their roles in preventive and integrated care delivery. This policy congruence constituted a critical enabling condition, fostering partnerships between Singapore General Hospital (SGH) and AACs. Influenced by these external drivers and motivated by local community norms and policy targets, AACs demonstrated increased openness to innovation and collaborative models, enhancing cosmopolitanism through strengthened inter-organizational linkages across tertiary care, community services, and government agencies.

Implementation was further facilitated by diversified funding streams, including philanthropic contributions from the Temasek Foundation and in-kind support such as staff time and venue provision by AACs, mitigating resource constraints and institutional burden. Community engagement activities, including focus groups with older adults, revealed high receptivity toward health promotion interventions, indicating strong congruence between community expectations and program objectives. Collectively, these factors underscore the outer setting’s critical role in establishing implementation readiness for ISHW.

#### Inner setting

The ISHW program was implemented within the inner setting characterized by AACs—community-based organizations distributed across Singapore with established infrastructure and workforce capacity to support social and health services targeting older adults. These centers possessed essential structural characteristics, including physical facilities, trained personnel, and operational routines for group-based engagement.

The program capitalized on existing coordination frameworks between AACs and SGH, integrating novel screening, nutritional counseling, and physical activity components into routine practice. It also enhanced information infrastructure through standardized screening instruments, personalized feedback reports, and continuous data exchange mechanisms.

Relational networks within AACs facilitated intersectoral collaboration with SGH via shared communication platforms, regular debriefings, and site visits, enabling effective real-time coordination and knowledge transfer.

Organizational culture within AACs was characterized by recipient-centered values emphasizing personalized service delivery, alongside a learning-centered orientation supporting ongoing staff development and reflective practice. Staff exhibited readiness for change, driven by recognition of service gaps in preventive health for older adults.

The ISHW intervention demonstrated high compatibility with AAC mandates (11) under the Age Well SG framework and was perceived as a priority initiative contributing to both local service credibility and national health outcomes. The alignment of mission and values between SGH and AACs was evident throughout the planning and implementation phases.

Critical enabling resources included staff availability, community venues, and training materials developed by SGH. These were supplemented by robust access to knowledge and expertise through on-site coaching, formal training modules, and implementation guides, collectively supporting program delivery.

#### Individuals

Implementation involved multiple stakeholder levels with differentiated yet interrelated roles. Senior leadership at SGH provided institutional endorsement and facilitated cross-departmental alignment with organizational objectives. Mid-level leaders, including clinical champions and program coordinators, managed operational planning, supervision, and troubleshooting, maintaining close engagement with AAC partners.

Within AACs, opinion leaders such as center managers and experienced volunteers played pivotal roles in fostering internal acceptance and motivating staff engagement with the innovation. Implementation facilitators from SGH—including physiotherapists and dietitians—delivered structured training and ongoing mentorship to AAC staff and volunteers, enhancing capacity and confidence.

Designated implementation leads, including the ISHW program coordinator and clinical leads, assumed responsibility for oversight of planning, execution, and quality assurance processes. The multidisciplinary implementation team encompassed experts from geriatrics, dietetics, physiotherapy, and health services research, contributing across design and delivery stages.

At the operational level, AAC staff and trained volunteers served as innovation deliverers, conducting screenings, facilitating physical activity sessions, and leading nutrition education. Their efficacy was contingent upon tailored capacity-building interventions and iterative feedback. Innovation recipients—community-dwelling older adults—actively contributed to program adaptation through participatory pre-implementation focus groups and ongoing session evaluations, ensuring responsiveness to user needs.

#### Process

The ISHW program employed a phased and participatory implementation process. Early collaborative planning sessions convened representatives from SGH and AACs, establishing cross-functional implementation teams.

Comprehensive needs assessments targeted both innovation deliverers (e.g., training requirements for AAC staff) and recipients (e.g., nutritional behaviors, motivational factors for physical activity). These assessments informed context-specific analyses, identifying organizational strengths and barriers.

Program design was iterative, incorporating workshops culminating in a logic model grounded in the Theory of Change and the Consolidated Framework for Implementation Research (CFIR). Tailored program materials and delivery strategies were co-developed with AAC stakeholders, exemplifying contextually adapted implementation.

Engagement strategies encompassed both staff and participants: AAC personnel were involved through co-creation workshops and structured training, while older adults contributed via focus groups and ongoing feedback mechanisms, reinforcing bidirectional engagement.

Implementation proceeded in a staged manner, initiating with two AACs to assess fidelity, optimize delivery, and build momentum prior to broader scaling. Reflection and evaluation were embedded continuously, incorporating stakeholder interviews and participant feedback, which informed iterative refinements such as enhanced exercise instruction clarity and supplementary take-home materials.

The program exhibited strong adaptive capacity, modifying content and delivery modalities in response to emergent needs—e.g., tailoring physical activity sessions to accommodate mobility variations and customizing nutrition modules to align with cultural dietary preferences.

#### Innovation

The ISHW program’s primary innovation resides in its modular design, enabling flexible implementation of core components—screening, nutrition, and physical activity—as integrated or discrete interventions contingent upon site-specific readiness and resource availability. This modularity facilitates scalable integration of the WHO ICOPE framework within Singapore’s decentralized community health infrastructure.

Capability-building strategies were similarly modular and responsive. For example, physiotherapists developed a competency-based training checklist to evaluate AAC staff proficiency in delivering physical activity interventions, linking identified gaps to targeted modular training sessions. Dietitians employed focus groups to ascertain nutrition knowledge deficits among trainers and resident needs, informing adaptive content development.

This modular approach mitigated complexity inherent in multi-domain interventions implemented within resource-limited community settings. Delineating discrete components with dedicated tools and training pathways simplified operationalization and allowed incremental adoption.

Program materials—including standardized checklists, visual aids, and “train-the-trainer” toolkits—were co-designed with end-user input and iteratively refined through pilot testing, enhancing usability and fostering stakeholder ownership.

Cost-effectiveness was enhanced through selective deployment of program components tailored to feasibility and relevance, circumventing a one-size-fits-all paradigm and leveraging existing assets such as trained personnel and ongoing group activities.

Collectively, the ISHW program exemplifies innovation via its adaptable, modular, and context-responsive design, addressing known barriers to integrated care implementation for older adults and supporting sustainability and scalability within diverse community care environments.

## Understanding key programmatic elements

### Theory of Change methodology

The Theory of Change (ToC) (8) is useful for developing and evaluating complex programs as it maps the steps to achieve intended outcomes, identifies enablers and barriers, and clarifies stakeholder roles. By applying ToC and CFIR methodologies, the program aims to provide a replicable, context-adaptable framework for scaling similar initiatives in aging populations globally.

#### Phase 1: Needs analysis

##### Methods

Before launching the ISHW program, a needs analysis was conducted with AACs to identify gaps in the existing system and inform targeted improvements. Initial interviews were conducted with residents of the AACs to identify preferred features that they would like to see in the physical activity and nutrition programs. Participants were recruited by AAC staff using purposive sampling to ensure representation across gender and activity levels. Verbal consent was obtained and no identifiable data were collected. A total of 20 residents were interviewed in 5 focus groups. The participant pool consisted of 5 male and 15 female participants, reflective of the typical AAC demographic. The ages of interviewed residents ranged from 64 to 85 years of age, which was representative of the demographics of residents that frequent AACs. The qualitative analysis of the interviews was led by XH (ISHW clinical lead) and thereafter reviewed by WQY (ISHW program coordinator), XX and HYT (researchers from SGH’s Health Services Research Unit [HSRU]). Data were analyzed in Excel using codebook thematic analysis following Boyatzis’ (12) structured code-development framework Initial codes were generated inductively from participant responses and iteratively refined using predefined domains relevant to program components (screening, nutrition, and physical activity). Four researchers (XH, WQY, XX, HYT) independently reviewed transcripts and applied preliminary codes, followed by consensus meetings to resolve discrepancies and establish reliability through negotiated consensus, thus enhancing reliability. Manual coding was conducted using Microsoft Excel. AAC staff, who were also trainers, joined in on the discussions as well and agreed with the points raised by residents during the interviews. Furthermore, trainer perspectives were inferred from residents’ perceptions of instruction style, and triangulated with input from AAC staff during co-design and feedback workshops. This allows the clinical and social aspects of themes to be identified from both the trainers’ as well as the residents’ perspectives.

Credibility was strengthened through analyst triangulation (four coders, two disciplinary backgrounds) and member-checking of preliminary themes with AAC staff. Transferability was supported by thick descriptions of the AAC setting, participant demographics and program context, enabling readers to assess relevance to similar settings. Dependability was ensured through systematic documentation of the coding process, with regular peer debriefings to discuss analytic decisions. Confirmability was supported by Excel documents and reflexive memos that were circulated among the coding team during weekly peer-debrief sessions.

#### Results of needs analysis

##### Physical activity program

Themes related to participants’ prior experiences, ideal program structure, and factors influencing sustained participation were identified. When interviewed, participants described the previous exercise programs at AACs as overly prescriptive, often limited to routines delivered via television. Thematic analysis identified subthemes of low motivation stemming from lack of personalized guidance, perceived low rigor of activities, and limited variety in exercises.

A participant noted dissatisfaction with previous activities, stating, “*He doesn’t demonstrate, and we didn't like it very much*.”

Conversely, many participants highlighted benefits of regular group exercise, with the overarching theme being its positive impact on overall health. Subthemes included increased physical strength, enhanced opportunities for social interaction, and improved self-motivation to engage in consistent physical activity.

One participant remarked, “*If you learn these things, you have to sweat … when you sweat, [that means] you have exercised … the body will be well*.”

Regarding program structure, participants expressed a preference for supervised, structured sessions that provided guidance on proper form and techniques. These participants appreciated rigor in instruction and valued assessments at the end of the program to evaluate their progress.

As one participant explained, “*It’s better to have an exam because you know what you’ve learned,” adding that such assessments allowed them to “[s] ee if you have learned and improved*.”

To encourage sustained participation after program completion, participants stressed the importance of follow-along resources, such as printed guides or online videos, to facilitate independent exercise. These resources were seen as essential for maintaining physical activity outside of structured group settings.

The emphasis on group-based activities, or “exercise together,” promotes physical health improvements while fostering social connections among participants. This approach addresses both the physical and social dimensions of wellbeing.

##### Nutrition program

Through understanding of the landscape and initial engagement with AAC operators, the program team identified nutrition education initiatives at AACs were scarce and tended to run on an ad-hoc basis. In our needs analysis done above, the ISHW team inquired community-dwelling older adults’ baseline nutrition knowledge on healthy eating, how do they apply existing nutrition knowledge into their daily lives, dietary misconceptions, ways of seeking nutrition information and self-motivation, preferred mode of learning and nutrition topics.

The team found that participants practiced food portion control, dietary restrictions (i.e., no added salt, oil, sauces, sugars in beverages), balance intake from each food groups to achieve diet quality and use of healthy cooking methods.

As a participant mentioned, “*Portions must be right, go by the plate-*Another participant remarked when asked regarding home cooking practices, *“[I] boil a pot of soup then I do not add salt.,” whilst another shared “Steaming is much more healthier.”*

Key barriers to maintaining healthy eating included social pressures during gatherings, the influence of family and friends, the limited availability of healthier food options when dining out and cost of foods, where nutritious foods were seen as expensive.

A participant commented, *“If you want nutritious foods, they are always very expensive….”*

Another shared, *“I try to have balanced diet, but I also indulge in Macs when I follow my children. If I have a choice on my own, I will try to take less rice, more balanced diet [consisting of] fish, meat.”*

Conversely, participants identified potential enablers, such as structured nutrition education programs inclusive of interactive, hands-on activities and knowledge-sharing platforms were their preferred mode of learning, enabling them to make informed choices.

As one participant shared, *“Hands-on is better,… at least people learn and know how to cook.”*

Focus groups were particularly instrumental in clarifying causal pathways and ensuring that proposed program activities (e.g., group exercises, nutrition education) would lead to the intended intermediate outcomes (e.g., increased health literacy, sustained behavioral change). For example, it is assumed that older adults would participate consistently if offered community-based physical activity programs. The focus group found that in addition, participants preferred guided sessions and requested follow-along materials for independent exercise. This prompted the inclusion of “exercise guides” as part of the output in the logic model below.

Similarly, in the nutrition domain, assumptions around knowledge gaps were explored. Residents showed partial knowledge of healthy eating but cited barriers such as affordability and family influence. These findings reinforced the importance of hands-on, culturally relevant nutrition sessions and shaped program outputs (e.g., communal dining with facilitated discussion) and assumptions (e.g., family context as a mediating factor).

This iterative dialogue aligns with Breuer’s view that ToC refinement should be evidence-based and responsive to community insight, which increases the likelihood of implementation success and sustainability.

Following the development of the ISHW Program’s ToC, the next phase involved translating these conceptual pathways into a structured, implementation-focused logic model. This was achieved through an iterative, stakeholder-informed process, aligning with Breuer et al. (13) ToC checklist, which emphasizes participatory development, iterative testing of causal assumptions, and alignment of interventions to context and outcomes.

Critically, the transition from ToC to logic model was not purely linear but dynamically informed by CFIR which served as an overarching lens for understanding how contextual factors and system readiness influenced program design and delivery. The CFIR-ToC mapping process involved structured consensus workshops with the ISHW team (clinicians, researchers, AAC staff) to align identified implementation barriers with hypothesized causal pathways and planned outputs.

###### Logic model development

####### Phase 2: Co-design and logic model development

**Figure 1:**
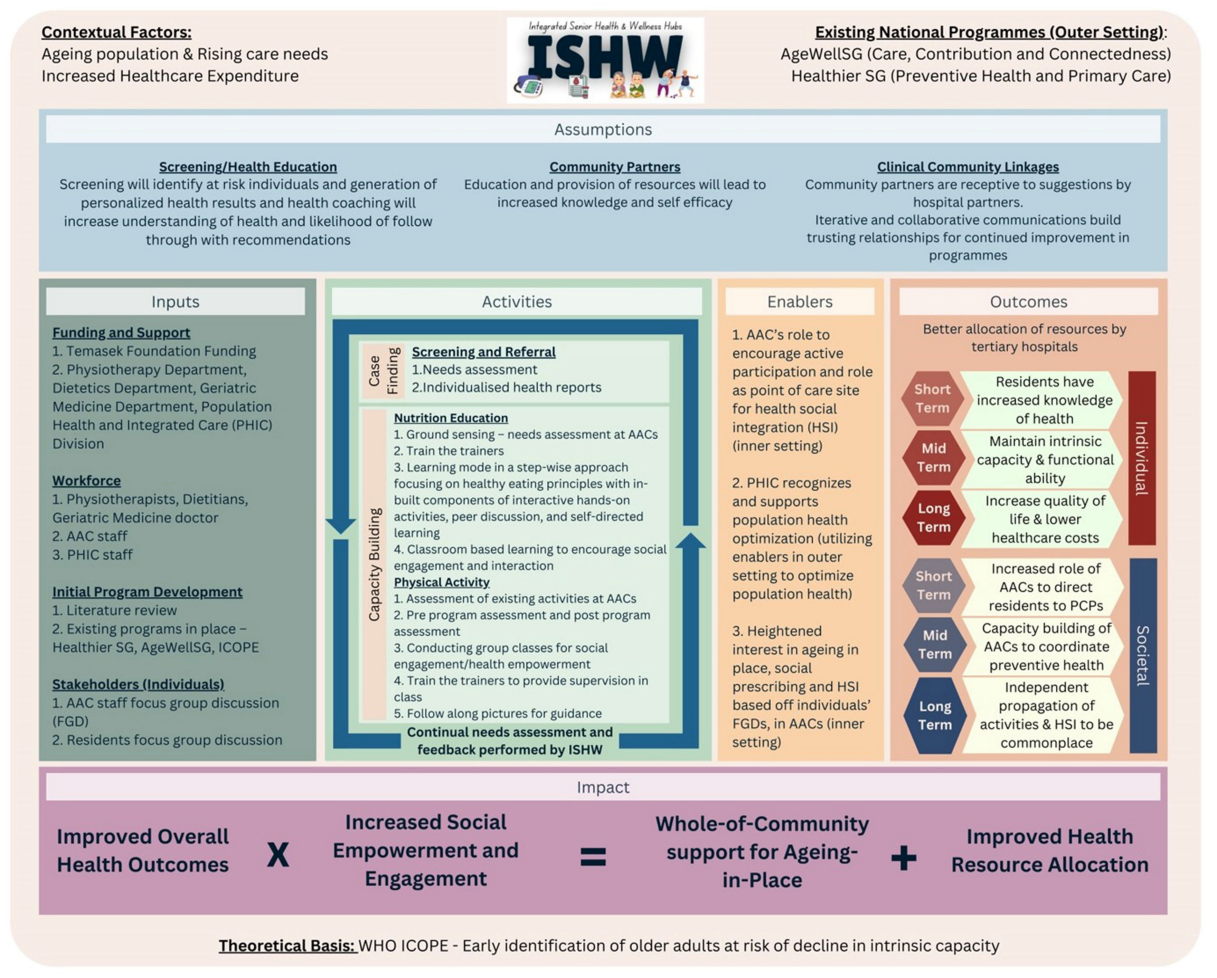
Final logic model for the ISHW program.

The ToC was operationalized into a logic model through structured workshops, incorporating CFIR-informed insights and community needs analysis. Three co-design workshops engaged 12 stakeholders (6 SGH clinicians, 4 AAC managers, 2 implementation researchers). Participatory logic model building and validation of CFIR constructs were used to refine program design. Workshop flip-charts and field notes were summarized into an Excel template, then coded deductively to the evolving logic-model components (inputs, activities, outputs, outcomes). Analytic memos captured disagreements with creation of successive logic-models before the final version in Figure 1. To grant further clarity, we mapped the CFIR domains to specific elements of ISHW in Table 1. With the AgeWell SG and HealthierSG policies framing the healthcare landscape, community partners are receptive to suggestions from tertiary hospitals to better the health of their residents. As AACs are tasked with the goal of encouraging health and social integration within the community, they are also keen to increase their own knowledge and self-efficacy by partnering with tertiary hospitals. Through needs analysis of participants and tailoring programs to their needs, personalized health results and coaching increases understanding of their health and following through with recommendations.

**Table 1:** Mapping of CFIR to elements of ISHW program.

CFIR domain	Construct	Element of ISHW program
Innovation	Innovation source	Internally developed by SGH with co-design involving AACs and adapted from WHO ICOPE
	Innovation evidence base	WHO ICOPE framework and supporting literature on healthy aging guided design; evidence-based screening and interventions
	Innovation relative advantage	Provides integrated, community-based preventive care vs. fragmented traditional care
	Innovation adaptability	Program tailored to AAC contexts; screening, physical activity, and nutrition sessions adapted to resident feedback
	Innovation trialability	Piloted in two AACs, iteratively refined before expanding to more sites
	Innovation complexity	Multi-component across sectors (screening, diet, physical activity); simplified via standard protocols and train-the-trainer model
	Innovation design	Packaged into screening toolkits, exercise guides, training modules; visually and operationally user-friendly
	Innovation cost	Funded by Temasek Foundation; uses existing infrastructure to reduce burden
Outer setting	Critical incidents	Launch of Healthier SG and Age Well SG catalyzed institutional interest and receptivity
	Local attitudes	Older adults showed enthusiasm for group exercise and nutrition sessions when properly tailored
	Local conditions	AACs have space, staff, and recurring programs, facilitating implementation in a community context
	Partnerships and connections	Strong cross-sector partnerships: SGH, AACs, public health agencies
	Policies and laws	National aging policies (Healthier SG, Age Well SG) align with program goals, encouraging support
	Financing	Funded through grant support and leveraging SGH and AAC resources
	External pressure	Government emphasis on aging in place, performance monitoring under Age Well SG; community expectations of AAC services
Inner setting	Structural characteristics	AACs offer physical space, program staff; SGH provides clinical oversight and technical support
	Relational Connections	Strong partnerships between SGH clinicians and AAC teams. AACs serve as community hubs with embedded partnerships with tertiary care institutions (SGH)
	Communications	Regular feedback loops, co-design sessions, and training workshops ensure continuous communication
	Culture—human equality centeredness	Inclusive recruitment and adaptation for all older residents regardless of background
	Culture—recipient centeredness	Needs assessments and co-creation ensured alignment with older adults’ values
	Culture—deliverer centeredness	AAC staff trained and empowered to deliver content with contextual sensitivity
	Culture—learning centeredness	Strong emphasis on learning and iteration (train-the-trainer, reflection loops)
	Tension for change	AACs identified lack of structured, evidence-based programs; ISHW addressed this unmet need
	Compatibility	Fully compatible with AAC workflows, staff capacity, and national goals
	Relative priority	High priority due to alignment with national mandates and AAC operational goals
	Incentive systems	Non-financial: professional development, visibility, recognition from health authorities
	Mission alignment	Strong congruence with AAC and SGH goals of integrated aging care.
	Available resources	AACs provided venue/staff; SGH provided training, materials, and clinical support
	Access to knowledge and information	Staff and volunteers received structured training and follow-up materials
Individuals	Leaders (high, mid and opinion leaders)	SGH leadership provided strategic direction and support for scaling. SGH clinical leads coordinated pilot and supported AAC engagement. Senior AAC managers and influential volunteers helped advocate for program within centers
	Implementation (leads, facilitators and team members)	Program coordinator and clinical leads oversaw planning, delivery, and quality. SGH staff acted as facilitators through mentoring and on-site coaching. Multidisciplinary team from SGH including dietitians, physiotherapists, doctors
	Innovation deliverers	AAC staff and trained volunteers executed screening, nutrition, and physical activity components
	Innovation recipients	Community-dwelling older adults aged 60 years and older, engaged in structured program activities
Implementation	Teaming	Multidisciplinary collaboration between SGH clinical staff and AAC operators
	Assessing needs—innovation deliverers	AAC staff needs assessed via focus groups and capability review
	Assessing needs—innovation recipients	Older adults engaged in focus groups to identify preferences and barriers
	Assessing context	AAC readiness and infrastructure reviewed during program co-development
	Planning	Co-design process included logic model development, curriculum design, pilot sequencing
	Tailoring strategies	Program customized for each AAC; adapted delivery based on resident feedback
	Engaging—innovation deliverers	AAC staff engaged via training, coaching, and participatory planning
	Engaging—innovation recipients	Older adults involved via focus groups, feedback on sessions, and interactive formats
	Reflecting and evaluating—implementation	Ongoing qualitative feedback and stakeholder interviews used to refine delivery
	Reflecting and evaluating—innovation	Focus group input refined exercise and nutrition modules; assessments guided content changes
	Adapting	Program continuously iterated through ToC refinement, local customization, and stakeholder feedback

ToC guided the translation of CFIR-informed strategies into a structured implementation plan. By mapping inputs such as training resources and partnerships to specific activities like community screenings and guided exercise sessions, the program was able to define measurable outputs such as the number of staff trained and participants engaged. This ensured that program efforts were aligned with the broader goal of improving functional ability and social participation among older adults.

With the final logic model in place (Figure 1), we next discuss the key findings from the program development process, highlight its alignment with policy frameworks, and compare ISHW’s design to other ICOPE implementations globally.

## Discussion

This study successfully achieved its aim of developing and refining an implementation-informed logic model for the ISHW Program by applying CFIR and ToC methodologies to integrate the WHO ICOPE framework into a community-based, health-social care model.

### Integrated approach

The ISHW Program adopts an integrated approach, blending health initiatives like chronic disease screenings and physical activity and nutrition education with social engagement to promote active, connected aging. By integrating social engagement across nutrition and exercise domains, ISHW addresses both health outcomes and social isolation—a key determinant of older adult wellbeing.

The ISHW Program aligns with both global and national frameworks on healthy aging. It operationalizes WHO ICOPE principles by enhancing intrinsic capacity and functional ability through a person-centered, community-based model. Concurrently, it supports national initiatives such as Healthier SG and Age Well SG by leveraging AACs as delivery hubs for preventive care.

This alignment strengthens implementation readiness, enabling the program to meet emerging policy goals while addressing local health and social needs in a coordinated manner.

### Strengths of the ISHW program

In addition to aligning with ICOPE-specific implementation efforts, the ISHW Program reflects core principles of implementation science described in broader public health literature. Implementation frameworks can be classified into three types: process models, determinant frameworks, and evaluation frameworks. The ISHW approach integrates elements from all three—CFIR as a determinant framework, ToC as a process model, and the logic model as a planning and evaluation tool—demonstrating its robustness in guiding real-world implementation.

Globally, several programs have adopted logic models or ToC frameworks to guide the implementation of the WHO ICOPE approach. These frameworks enable planners to align complex interventions with intended outcomes and provide mechanisms for iterative refinement and stakeholder engagement.

For instance, the ICOPE France pilot (2) highlighted feasibility of early screening and multidisciplinary collaboration but identified workforce shortages and training gaps as sustainability barriers. Anticipating these challenges, the ISHW Program embedded a train-the-trainer model to build community capacity for long-term delivery.

In Hong Kong (3), readiness assessments using ToC revealed that intersectoral collaboration, workforce competency, and stakeholder trust were key to implementation success. ISHW reflects these lessons by incorporating participatory planning, community feedback, and co-development workshops to ensure contextual fit and stakeholder ownership.

A recent review (14) in the WHO ICOPE implementation toolkit emphasized the need for participatory logic modeling and integration of social dimensions in ICOPE-aligned interventions. The ISHW Program exemplifies this through its holistic design—combining physical activity, nutrition, and social cohesion strategies such as group meals and peer-led exercise, tailored to community preferences.

Brazil’s (15) ICOPE implementation similarly used logic modeling to build adaptability and program retention in low-resource settings. Their findings support ISHW’s emphasis on continuous refinement, particularly in response to local feedback and policy developments such as Age Well SG.

Overall, ISHW contributes to the growing body of implementation literature by offering a scalable, ToC-informed community intervention grounded in the ICOPE framework. The program distinguishes itself through:

- Comprehensive CFIR-ToC integration, allowing systematic identification of implementation barriers and enablers;
- Co-development with community partners, enabling high contextual relevance;
- Sustainability focus, with a structured model for staff and volunteer training.

These attributes demonstrate how participatory, theory-informed implementation strategies can enhance uptake, fidelity, and long-term sustainability of integrated care interventions for older adults.

### Methodological constraints

Several limitations warrant consideration. Firstly, the qualitative approach limits generalizability. While the findings offer rich contextual insights, future studies employing quantitative or mixed-method designs could enhance external validity Secondly, the program focused on older adults engaged with AACs, potentially excluding perspectives of socially isolated individuals or those with limited access to services. Further research is needed to explore their specific needs and implementation barriers. Thirdly, stakeholder feedback and logic model development were based on single time points. A longitudinal evaluation—currently being planned—will assess sustained engagement, behavioral change, and health outcomes, offering a more comprehensive view of program impact over time.

### Challenges and lessons learned

Initial iterations of the logic model (Supplementary Appendix 1) revealed gaps in activity specification, output mapping, and outcome definitions. These challenges highlighted the need for structured implementation frameworks to clarify program design.

Stakeholder input proved essential in refining the model. Feedback underscored the need for clearer coordination mechanisms and more tailored training for AAC volunteers. This led to enhanced training content and improved communication pathways between healthcare and community actors.

A key lesson was the value of iterative design. Regular stakeholder engagement allowed the program to remain adaptive, ensuring feasibility, contextual fit, and continued alignment with both community expectations and policy shifts.

### Implications for practice and policy

These findings offer important insights not only into program design, but also into broader lessons for health system integration and healthy aging policy. By integrating health interventions with social engagement, it addresses challenges of aging populations, such as managing health needs and reducing social isolation. This model is relevant for urban areas facing similar demographic trends and provides insights into community-based strategies for healthy aging.

The program’s adaptable design allows for scalability and sustainability, particularly in densely populated urban areas. It leverages existing infrastructure, such as AACs, and expertise from healthcare institutions like SGH, to deliver coordinated care. Its flexibility enables customization for different communities, making it viable for broader implementation.

With ongoing stakeholder engagement, the program could be scaled to serve larger populations locally and globally. By incorporating early screening, targeted interventions, and social interaction, it proactively addresses health issues and promotes functional ability, with potential to reduce reliance on acute healthcare services if sustained engagement leads to improved functional health to overall support healthier, independent aging.

### Future directions

The ISHW team aims to scale the program while maintaining stakeholder engagement and responsiveness. Ongoing feedback loops will ensure continued alignment with evolving national priorities and community needs. The planned longitudinal evaluation will assess long-term outcomes and inform refinements to the model.

Insights from ISHW will also inform broader health-social integration strategies, offering a blueprint for context-adapted, sustainable community-based aging interventions.

## Conclusion

In conclusion, this study aimed to develop and pilot a context-sensitive, implementation-informed model of integrated care for older adults, guided by the WHO ICOPE framework and structured using the Consolidated Framework for Implementation Research (CFIR) and Theory of Change (ToC). By applying a phased implementation approach and participatory co-design, the ISHW Program demonstrates how integrated health and social interventions can be operationalized in community settings, with potential for scalable and sustainable aging-in-place models. Its focus on preventive care, holistic wellbeing, and community-driven solutions marks a paradigm shift in aging care.

## Data Availability

The raw data supporting the conclusions of this article will be made available by the authors, without undue reservation.
