# Supplementary material for: From the WHO framework to integrated senior health and wellness hub program: an implementation journey
Source: Front Public Health. 2025 Jul 28;13:1593490. doi: 10.3389/fpubh.2025.1593490 (PMC12336022; doi:10.3389/fpubh.2025.1593490)
Supplement: Supplementary file 1 [file Data_Sheet_1.docx]

# Appendix 1: Initial Logic Models

### 1. Initial Logic Model


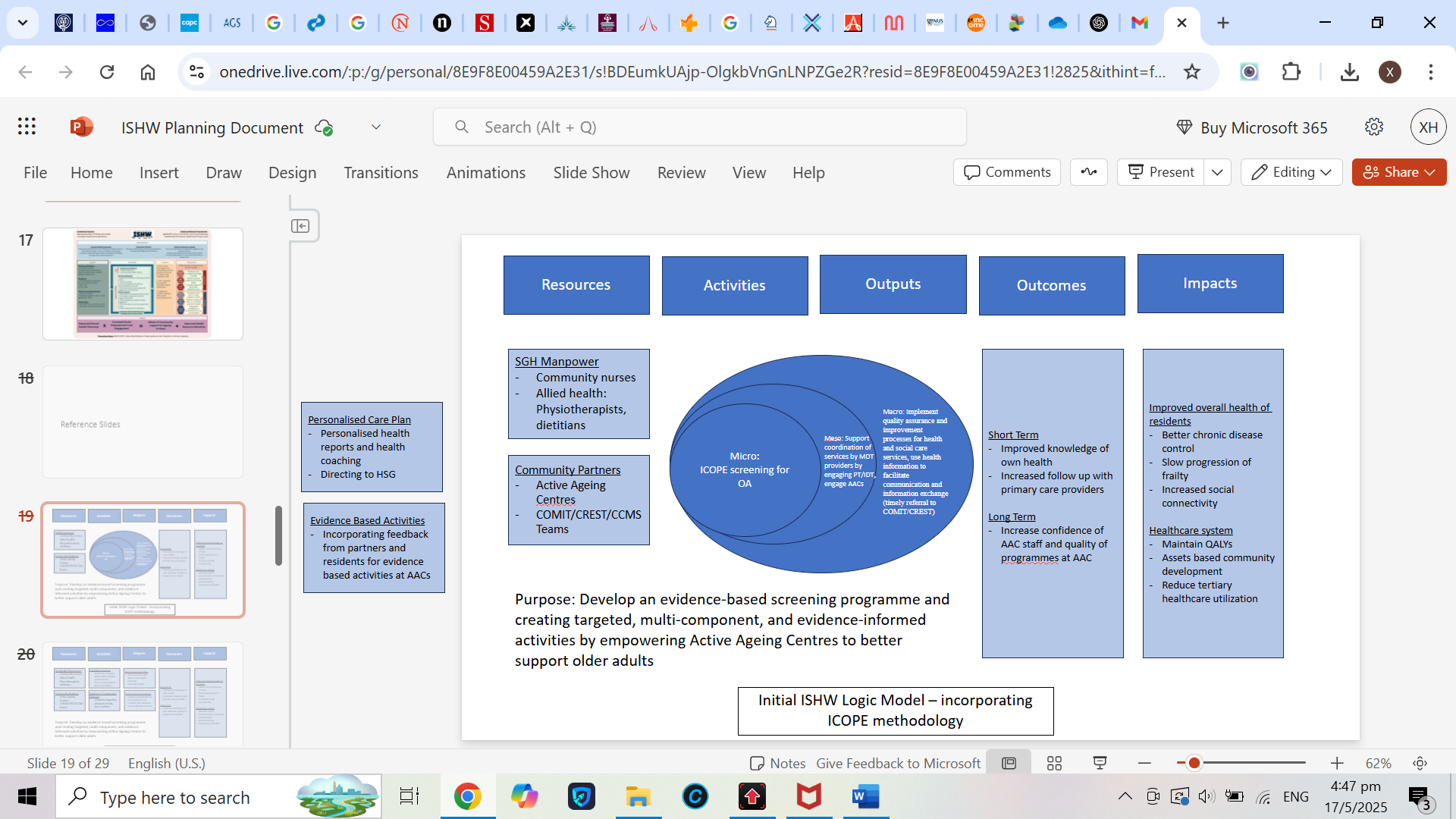


*Figure 1: Initial ISHW Logic Model*

The development of the initial logic model was guided by the principles of the ICOPE methodology, providing a structured starting point for conceptualizing the ISHW Program.

While it served as a foundational framework, the initial version exhibited several critical gaps that required further refinement:

1. Lack of Clarity and Specificity: The model lacked detailed descriptions of the specific activities to be undertaken and the outputs expected. Without this clarity, it was difficult to delineate the precise steps required for implementation or to gauge the immediate results of these activities.
2. Unclear Outcomes: The expected outcomes of the program were not well-defined, making it challenging to connect the program’s interventions to its overarching goals. This absence of clearly outlined outcomes hindered the ability to evaluate the program’s effectiveness or to measure its progress meaningfully.
3. Other Areas of Deficiency: Additional areas requiring improvement included the absence of medium- and long-term benchmarks, inadequate representation of stakeholder roles and contributions, and limited consideration of potential barriers and enablers that might influence the program’s success.

   Addressing these deficiencies in subsequent iterations was critical to creating a more comprehensive and actionable logic model, capable of guiding the successful implementation and evaluation of the ISHW Program.

### 2. Revised Logic Model


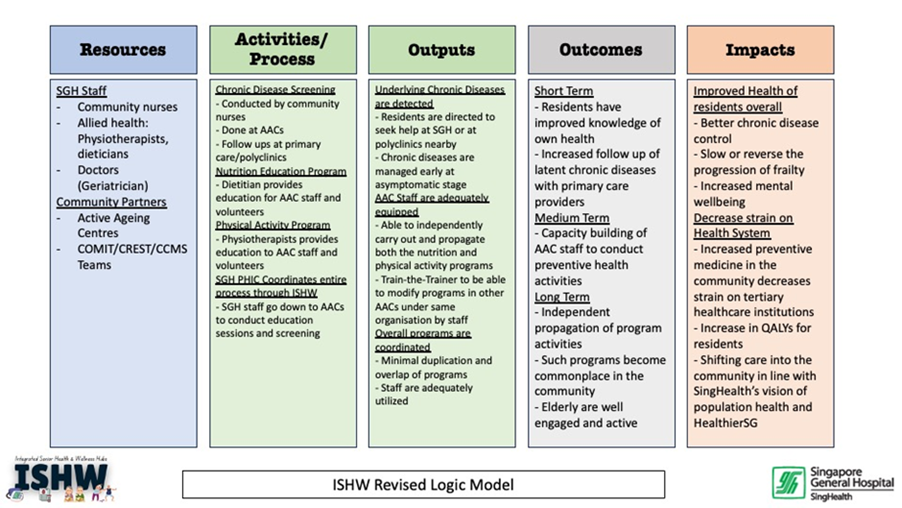


*Figure 2: Revised Logic Model*

Key updates were made to the "Activities" and "Outputs" sections, incorporating additional details to clarify the specific actions required and their immediate results. These refinements were informed by the feedback gathered during the workshop, ensuring that the logic model reflected both the program fidelity and adaptation as well as the diverse perspectives of stakeholders.

A significant enhancement was the inclusion of medium-term outcomes as an additional layer within the model. This addition provides a mechanism to evaluate whether the program is progressing towards its overarching goals in the medium term. By tracking these outcomes, the revised logic model allows for more dynamic monitoring of the program's effectiveness, enabling adjustments and improvements during implementation. This iterative approach ensures that the ISHW Program remains responsive to emerging challenges and opportunities, ultimately strengthening its impact and sustainability.

| **No.** | **Item** | **Description *(as defined by SRQR)*** | **Section / Location in Manuscript** |
| --- | --- | --- | --- |
| 1 | Title | Indicate that the study is qualitative (approach or data type) | Title page |
| 2 | Abstract | Summary of background, purpose, methods, results, conclusions | Structured Abstract |
| 3 | Problem formulation | Description of problem / phenomenon under study and significance | Introduction – Rationale |
| 4 | Purpose / research question | Specific objectives or research questions | Introduction – Study Objectives |
| 5 | Qualitative approach & research paradigm | Methodological orientation (e.g., case study) and guiding framework | Methods – Study Design (“community case study, informed by CFIR”) |
| 6 | Researcher characteristics & reflexivity | Researchers’ backgrounds, relationships to study, reflexivity practices | Methods – Reflexivity paragraph |
| 7 | Context | Setting / site and salient contextual factors | Context of Innovation section |
| 8 | Sampling strategy | How and why participants/documents were selected, saturation | Phase 1 Participants & Phase 2 Participants paragraphs |
| 9 | Ethical issues | Ethics approval, consent procedures, confidentiality | Ethics paragraph |
| 10 | Data-collection methods | Types of data, procedures, timeline | Phase 1: Needs Analysis – Data Collection |
| 11 | Data-collection instruments & technologies | Interview guides, forms, devices, modifications | Same paragraph (“semi-structured guide; flip-charts; Excel logs”) |
| 12 | Units of study | Number and relevant characteristics of participants / documents | Phase 1 Participants (n = 20 residents) & Phase 2 Participants (n = 12 stakeholders) |
| 13 | Data processing | Transcription, data entry, management, security | Methods – Data Management |
| 14 | Data analysis | Analytic process, coding, theme development, software | Phase 1 Analysis & Phase 2 Analysis using Excel codebook |
| 15 | Techniques to enhance trustworthiness | Credibility, transferability, dependability, confirmability strategies | Last paragraph of Methods |
| 16 | Synthesis & interpretation | Main findings / themes and their meanings | Results sections (Phase 1 & Phase 2) |
| 17 | Links to empirical data | Quotations, excerpts, field-note examples supporting themes | Results – inline exemplar quotes (pp 9–11) and Table 2 |
| 18 | Integration with prior work | How findings relate to earlier studies / theory | Discussion – Comparison with literature |
| 19 | Limitations | Trustworthiness and methodological constraints | Discussion – Limitations |
| 20 | Conflicts of interest | Potential sources of influence or perceived bias | Declarations – Conflicts |
| 21 | Funding | Financial support and role of funder | Declarations – Funding |

*Table 1: Standards for Reporting Qualitative Research Checklist*
